# Work‐Related Quality of Life and Well‐Being of Speech and Language Therapists in Ireland

**DOI:** 10.1111/1460-6984.70090

**Published:** 2025-07-25

**Authors:** Victoria Hogan, Mary Pat O'Malley, Michael Hogan, Margaret Hodgins

**Affiliations:** ^1^ Discipline of Health Promotion, School of Health Sciences University of Galway Galway Ireland; ^2^ Discipline of Speech and Language Therapy, School of Health Sciences University of Galway Galway Ireland; ^3^ School of Psychology University of Galway Galway Ireland

**Keywords:** Irish speech and language therapists, organisational constraints, quality of working life, turnover intention, well‐being, workload

## Abstract

**Background:**

Few studies have examined well‐being among speech and language therapists (SLTs), and no study to date has examined quality of working life. Research is also needed to better understand how individual and organisational factors influence quality of working life and well‐being of SLTs.

**Aims:**

The aims of this study were to characterise quality of working life and well‐being in Irish SLTs and determine the impact of individual and organisational factors on quality of working life and well‐being.

**Methods:**

SLTs in Ireland (*N* = 105) completed a cross‐sectional, electronic survey in the summer of 2022 which included measures of work‐related quality of life, organisational constraints, well‐being, workload and turnover intentions.

**Results:**

Well‐being and quality of work‐life scores were lower than published norms. Higher workload and higher organisational constraints were both related to lower quality of working life, *F*(4, 104) = 21.904, *p* < 0.001, and higher organisational constraints were related to lower well‐being, *F*(1,103) = 20.269, *p* < 0.001. Lower quality of working life in turn predicted higher turnover intention, *F*(1,103) = 31.502, *p* < 0.0005.

**Conclusions and Implications:**

The results indicate the importance of organisational constraints in relation to both quality of work life and well‐being of Irish SLTs. Workload is also an important factor related to quality of work life. Lower quality of working life is a problem for the SLT profession as it relates to higher turnover intention. This study demonstrates the importance of exploring the work experience of SLTs and highlights working conditions that may lower quality of working life and result in SLTs leaving the sector.

**WHAT THIS PAPER ADDS:**

*What is already known on this subject*

- Although QoWL has been widely examined in the healthcare sector, there has been very little examination of the QoWL of SLTs. Similarly, few studies have been conducted examining the well‐being of SLTs.

*What this study adds to the existing knowledge*

- This is the first study to examine QoWL and well‐being in Irish SLTs. QoWL and well‐being levels are low when compared to normative data in healthcare workers. Higher organisational constraints and higher workloads were related to lower QoWL, and higher organisational constraints were also related to lower well‐being. Lower QoWL in turn was related to higher turnover intentions.

*What are the actual and clinical implications of this work?*

- QoWL and well‐being for Irish SLTs are lower than expected and compare unfavourably with other healthcare professionals. Constraints faced by SLTs in their day‐to‐day work must be examined in order to alleviate the potential for stress and poor well‐being. Workload in particular will require resourcing in order to improve QoWL.

## 1 Introduction

Although it is a well‐established finding that health care workers (HCWs) experience higher levels of stress and burnout than many other occupational groups (Kinman 2021), there is a renewed focus on the well‐being of HCWs in the context of the extreme strain on health and social care services during COVID‐19. Much of this work focuses on physicians, nurses, and social workers. Speech and language therapists (SLTs) are a relatively understudied occupational group when it comes to understanding stress and well‐being, particularly within the context of service disruption during COVID‐19 and the subsequent need to address backlogs on routine service delivery (Müller et al. 2023). Although constructs such as job satisfaction and stress have been investigated in a limited number of studies of SLTs across a variety of countries (Ewen et al. 2021), the multi‐dimensional construct of Quality of Work Life (QoWL) has not been examined to date within the SLT profession, apart from studies where mixed allied healthcare professionals were included (see McFadden et al. 2020). Although SLTs have been included in existing allied healthcare studies, investigating the SLT profession specifically in relation to QoWL is an under‐researched area and therefore important to explore. Furthermore, reported rates of occupational stress among healthcare workers (37% of workers; Al Maqbali et al. 2024) and moderate forms of job burnout (15%–25% of EU workers; Aumayr‐Pintar et al. 2018) underline the importance of exploring QoWL in SLTs to inform planning for the development of a sustainable workforce (Smith‐Tamaray et al. 2021). Speech and language therapy services in Ireland experienced significant disruption as a result of COVID‐19, where changes to the working conditions and environment of SLTs were associated with tensions between demands and capacity (Müller et al. 2023). Consistent with findings from studies in the UK SLT service (Chadd et al. 2021), Müller et al. (2023) voice concerns about the negative impact on clients and families and the need for continued support for frontline healthcare workers, post‐pandemic, to ensure that neither service providers nor service users experience any further compromises to health and well‐being. As such, an investigation of the QoWL of SLTs in Ireland is not only necessary but timely. This article explores the broader construct of quality of work life, along with well‐being and turnover intentions in SLTs, adding to sparse literature on this topic. It also provides an insight into the post‐pandemic situation with a view to benefiting the profession, the health service, and its clients.

### 1.1 Quality of Working Life

The current study draws upon a socio‐ecological model of health which postulates that environments such as the workplace are important determinants of health (WHO 2010). This is consistent with work in the area of quality of working life, which highlights the conditions and practices in workplace organisations that can influence the health and well‐being of the workforce. Research on quality of work life extends back at least 60 years (Martel and Dupuis 2006) and includes multiple constructs, variables, and measures (Easton and Van Laar 2013). These broadly fall into two groups: (1) determining factors and (2) outcomes or effects. Much of the early work in the area focused on intrinsic determining factors such as socio‐technical features and work design and extrinsic factors such as remuneration, working hours, and working conditions; while in recent years researchers have extended the scope of research to include a focus on organisational issues such as resources, autonomy, control, and social support (Guest 2020). Frequently studied outcome variables include job satisfaction, self‐reported stress and general well‐being, and also worker involvement, enrichment and motivation (Easton and Van Laar 2013). Reviews of the construct of quality of working life (e.g., Easton and Van Laar 2018; Wallace et al. 2007), together, identify 33 different possible components, although overlap is evident. Many approaches divide components into those that are personal and those that are work‐related, the latter being amenable to system or service‐level intervention.

Although there is still no consensus in the literature on an exact definition of quality of working life, nor on the exact components that comprise it, QoWL has been examined widely in the healthcare setting. QoWL has been examined among nurses (Kaddourah et al. 2018), hospital employees (Mosadeghrad 2013), mixed allied HCW groups (McFadden et al. 2020), surgeons (Zubair et al. 2017), and occupational therapists (Hogan et al. 2023). There is evidence that QoWL is associated with turnover intention, for example, in nurses (Kaddourah et al. 2018), hospital employees (Mosadeghrad 2013), and occupational therapists (Hogan et al. 2023).

Governments and regulatory bodies are increasingly committed to improving worker well‐being in addition to their traditional focus on health and safety responsibilities. This is reflected in Ireland in the National Framework for Healthy Workplaces 2021–2025 (Ireland Department of Health 2021), a strategic plan to help maintain and improve worker health and well‐being. A renewed focus on QoWL as one means to improve employee well‐being (Grote and Guest 2017) highlights the need and opportunity to better characterise the multidimensional health challenges inherent in working environments. Easton and Van Laar (2018) have developed a model and measure of QoWL recommended for use with healthcare workers, designed to support theoretically driven investigations for the purpose of benchmarking and service improvement (Easton and Van Laar 2018). This model includes six factors: (1) general well‐being, (2) stress at work, (3) home‐work interface, (4) working conditions, (5) control at work, and (6) job and career satisfaction. The model was used recently to examine the impact of the COVID‐19 pandemic on the QoWL of allied healthcare professionals (McFadden et al. 2020).

Pring et al. (2012) highlighted that much of what we know about the working lives and conditions of SLTs is anecdotal and therefore may not be fully representative. Pring and colleagues documented a need for more empirical investigation of the practices and experiences of SLTs. Ewen et al. (2021) recently summarised the existing research on well‐being, job satisfaction, stress, and burnout in SLTs between 1998 and 2018, finding only 15 quantitative and two qualitative studies conducted over this time period. This review found that job satisfaction and turnover intention have been most studied to date, with a lesser focus on stress, burnout, and well‐being. More recently, studies focused on SLTs have examined the impact of the COVID‐19 pandemic on stress and burnout (Marante et al. 2023), job satisfaction (Farquharson et al. 2022), and service delivery (Müller et al. 2023). Ewen et al. (2021) note that the stress and burnout findings are not conclusive, and more studies are required to examine well‐being in the SLT profession. In order to advance understanding, Ewen et al. (2021) recommend that individual and work factors be included in future studies, such as those incorporated in Easton and Van Laar's QoWL model.

### 1.2 Speech and Language Therapy in Ireland

Speech and language therapy services are provided through the statutory health services in Ireland, the Health Service Executive (HSE). As of December 2022, there were 2295 SLTs registered with CORU (the statutory regulator for health and social care professionals) in Ireland (CORU 2022). There are no reported statistics on gender diversity in SLT in Ireland. However, anecdotal evidence suggests figures in line with HCPC (2021) report of 96% female as reported for the United Kingdom. Prior to COVID‐19, the Strategic Workforce Planning and Intelligence Unit in the HSE commissioned an analysis of workforce demand 2019–2035. According to Keegan et al. (2022), an increase of between 101 and 118 additional speech and language therapists is required by 2035, which represents an annual increase over this period of between 2.1% and 3.3%. This projected increase is required to ensure the sustainability of operations in the acute hospital sector in the face of staff shortages and increasing numbers of older people in the Irish population.

The COVID‐19 pandemic brought unanticipated challenges to the health services, including the interruption of non‐urgent care, which had a considerable impact on SLTs (Chadd et al. 2021). Redeployment of personnel, capacity issues connected to COVID‐19 staff sickness, and increasing demand with new and rising caseloads throughout the national emergency were other reported challenges (Rouse and Regan 2021). During the first wave of the pandemic in the spring of 2020, 46% of SLTs reported some degree of redeployment, and although this had dropped to 38% by autumn 2020, the impact on services was still significant, with demand increasing across all settings (Müller et al. 2023). Furthermore, SLTs were required to pivot from face‐to‐face service delivery to tele‐health, for which they were unprepared (Kollia and Tsiamtsiouris 2021).

More recently, a national programme changing how disability services are delivered, ‘Progressing Disability Services for Children and Young People’ (PDS), has been launched with the aim of achieving a unified approach to delivering disability health services (Health Service Executive 2024). According to the IASLT's (2022) statement on the status of PDS, its members are identifying systemic failures in the roll out that are placing children and SLTs at risk, resulting in concerns relating to CORU registration and SLT scope of practice. Furthermore, the IASLT (2022) report that the failures of PDS have a direct impact on staff retention and recruitment in disability services.

## 2 Aims

The QoWL and well‐being of SLTs in Ireland have not been examined, therefore, the aim of this study was to explore QoWL and well‐being among Irish SLTs in order to characterise the workforce experience. In addition, we investigated the relationship between personal and work‐related variables and QoWL and well‐being. Finally, we examined whether QoWL is related to well‐being and turnover intention (defined here as ‘the desire to relocate or leave an organization to find a better job’; Lestari and Margaretha 2021, 166).

## 3 Methods

### 3.1 Participants

At the time of the study (2022), the population of SLTs working in Ireland was estimated at 2205 (i.e., those registered with CORU). However, contact details were not available through CORU; therefore, contact was made with the IASLT, who had approximately 1100 SLT members, which roughly equals 50% of the population of SLTs in Ireland. Inclusion criteria for the study stipulated that respondents be currently working as SLTs and have greater than 1 year's work experience.

### 3.2 Procedure

The questionnaire survey was distributed electronically using Microsoft Forms as the platform. The IASLT agreed to act as a gatekeeper for the study by sending the electronic questionnaire survey to its members on behalf of the research team. An email invitation to participate in the study was sent via the IASLT to its members. The email contained the study information sheet and the link to the electronic survey. All 1100 members of IASLT were invited to participate. In addition, links to the survey and information sheet were distributed by the Discipline of Speech and Language Therapy at the University of Galway using their social media accounts, specifically, using Facebook and X (formerly Twitter). Data collection occurred over a 3‐week period in June and July 2022. All participants provided informed consent via a mandatory consent question at the start of the electronic questionnaire. Ethical approval was granted by the University of Galway Research Ethics Committee in March 2022.

### 3.3 Questionnaire

A short questionnaire, taking less than 15 min to complete, was designed with reference to the questionnaire measures employed by McFadden et al. (2020). The questionnaire consisted of three sections:

- **Section 1** examined the demographic profile of respondents and included questions on age, gender, ethnicity, education, disability status, marital status, and caring responsibilities.
- **Section 2** asked 15 questions about respondents’ work characteristics, specifically, professional area of work, location in country, sector, job tenure and role, working hours/overtime, sick leave, COVID leave, and redeployment.
- **Section 3** of the questionnaire employed five validated subscale measures: (1) Quality of Working Life Scale, (2) WHO 5 Wellbeing Index, (3) Turnover Intentions Scale, (4) Quantitative Workload Inventory, and (5) Organisational Constraints Scale. Details on the five validated measures are provided below:
  1. The *Quality of Working Life Scale* (Van Laar et al. 2007) includes six factors: (1) General Well‐Being (six items), (2) Home‐Work Interface (three items), (3) Job and Career Satisfaction (six items), (4) Control at Work (three items), (5) Working Conditions (three items), and (6) Stress at Work (SAW) (two items). All scale items are rated using a five‐point Likert scale response option ranging from ‘Strongly Disagree’ to ‘Strongly Agree’. Scoring instructions for each factor and the overall scale score were followed (Easton and Van Laar 2018). Higher scores indicate better quality of work life.
  2. The *WHO Five Well‐Being Index* (WHO‐5; World Health Organisation) includes five questions that measure general well‐being, for example, ‘… my daily life has been filled with things that interest me’ (WHO 1998). A five‐point Likert scale is employed with responses ranging from ‘at no time’ to ‘all of the time’. Raw scores range from zero to 25, and the summed scale item scores are multiplied by 4 to provide a final score, which ranges from zero (absence of well‐being) to 100 (best imaginable well‐being) (Topp et al. 2015).
  3. The *Turnover intention Scale* (Michaels and Spector 1982) includes three scale items (e.g., ‘I intend to quit my current job’). A six‐point Likert scale is employed, ranging from ‘strongly disagree’ to ‘strongly agree’. Higher scores indicate greater turnover intentions.
  4. The *Quantitative Workload Inventory* (Spector and Jex 1998) consists of five questions. Respondents indicate how often situations occur, for example, ‘how often is there a great deal to be done?’ A five‐point Likert scale ranging from ‘less than once per month’ to ‘several times per day’ is employed. Higher scores indicate higher levels of workload (Spector and Jex 1998).
  5. The *Organisational Constraints Scale* (Spector and Jex 1998) consists of 11 items. Respondents indicate how often or difficult they find it to do their job because of the 11 items, for example, ‘Organizational rules and procedures’. A five‐point Likert scale ranging from ‘less than once per month’ to ‘several times per day’ is used for responses. The higher the score, the higher the level of constraints (Spector and Jex 1998).

### 3.4 Data Analysis

Descriptive data analysis was conducted and included calculation of means and standard deviations for continuous variables and frequencies and percentages for categorical level data. Average scores for the factors and overall score as measured by the Quality of Working Life Scale and overall scores on the Organisational Constraints Scale, Quantitative Workload Inventory and WHO‐5 measures were compared to normative data available for each instrument. This comparison establishes the level of functioning in these areas relative to other studies. Scale reliability was assessed by calculation of Cronbach alpha for the questionnaire measures. Regression analysis was employed to examine relationships between personal and work‐related variables and both QoWL and well‐being and also to examine whether QoWL is related to turnover intentions. Quantitative data was analysed using SPSS, Version 29.

## 4 Results

In total, 105 questionnaires were completed, which equates to an approximate response rate of 10% of the IASLT membership and (employing the CORU figure of 2205) 5% of the total workforce. All respondents to the questionnaire survey were female, with the majority aged between 18 and 49 years. Table 1 presents the demographic and work characteristics data for the sample. The majority identified as White Irish (98%), and most had completed undergraduate studies (67%), with the remainder having completed postgraduate studies. Very few participants indicated that they had elder‐caring responsibilities (8%), while a larger proportion had child dependents in the home (40%). Data in Table 1 indicates a range of years’ work experience within the sample, with the majority working full time and in the public sector.

**TABLE 1:** Demographic and work characteristics of participants.

Variable	*N* = 105	%
**Age**		
18–29	36	34
30–39	39	37
40–49	23	22
50–59	6	6
60–65	1	1
65+	0	0
**Marital status**		
Single	42	40
Married	44	42
Co‐habiting	19	18
Divorced/Separated	0	0
**Children**		
Yes	42	40
No	63	60
**Qualification**		
Postgraduate	35	33
Undergraduate	70	67
**Employment Status**		
Full time	87	83
Part time	18	17
**Sector**		
Public	90	87
Private	4	4
Voluntary/Community/Other	10	9
**Years of Work Experience**		
< 5	34	32
6–10	25	24
11–20	31	30
21–30	14	13
> 30	1	1

### 4.1 QoWL, Well‐Being and Turnover Intention

Table 2 presents the average scores and standard deviations for each of the six QoWL factors and overall score. Average scores on each factor were compared to the NHS UK norms (Easton and van Laar 2018). All QoWL factor scores and the overall score in this study were lower than the published norms. The overall score on the QoWL measure (2.97) is at the 20th percentile level, indicating that the average score in this study is equal to or higher than only 20% of NHS HCWs. The WHO‐5 average score (46.06) is below the cut‐off threshold of 50, indicating low average levels of well‐being among the sample, with 61% of the sample scoring below the cut off. Equally, the score on the General Well‐being component of the WrQOL measure puts the average score (3.34) at the 30th percentile level (Easton and van Laar 2018). A large proportion of the sample had considered resigning from their job (70%); however, just over a quarter of the sample (26%) indicated that they intended to quit, and 30% indicated that they were looking for other jobs. The average score on the turnover intentions scale is presented in Table 2. Cronbach's alpha is also presented in Table 2 for the QoWL factors and the other questionnaire measures. The reliability of the scales employed all exceeded .7, with most scales indicating very good levels of reliability.

**TABLE 2:** Means, standard deviations and Cronbach alphas on questionnaire measures.

	Mean	SD	Relationship with Norms	Cronbach's alpha
Work‐related Quality of Life	2.97	0.66	20th percentile	
1. General Wellbeing Factor (GWB)	3.34	0.73	30th percentile	0.860
2. Home‐work interface (HWI)	3.20	1.04	30th–40th percentile	0.834
3. Job Career Satisfaction (JCS)	3.14	0.73	30th percentile	0.782
4. Control at Work (CAW)	3.09	0.91	30th–40th percentile	0.721
5. Stress at Work (SAW)	1.95	0.91	20th percentile	Not calculated—only 2 items in scale
6. Working Conditions (WCS)	3.05	0.94	30th percentile	0.797
Turnover intention	10.79	4.34		0.827
WHO‐5 Well‐being	46.06	13.98		0.903
Organisational Constraints^*^	25.47	8.28	Higher than norm	
Quantitative Workload Inventory	20.96	4.31	Higher than norm	0.872

* *Note*: Calculation of Cronbach alpha is not advised (Spector and Jex 1998).

### 4.2 Organisational Constraints and Quantitative Workload

**FIGURE 1:**
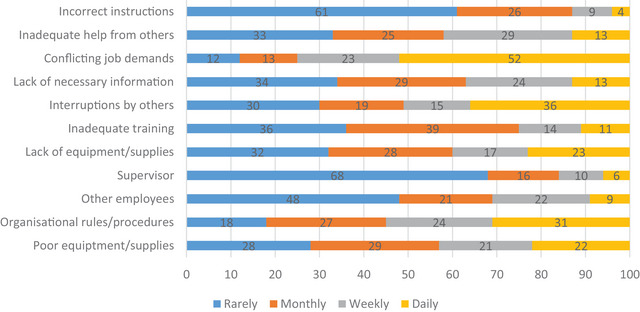
Distribution of responses to the items on the Organisational Constraints Scale presented in percentages. Figure 1: The distribution of responses to the 11 questions of the Organisational Constraints Scale by participants. Numbers within the figure indicate the percentage of the sample who reported the constraint. Colour coding of the bars indicates the frequency at which each organisational constraint was experienced. Blue indicates that a constraint was experienced rarely, orange indicates that a constraint was experienced on a monthly basis, grey indicates that a constraint was experienced weekly and yellow indicates that a constraint was experienced daily. For example, 52% of participants reported experiencing conflicting job demands on a daily basis whereas only 4% of participants reported receiving incorrect instructions on a daily basis.

Figure 1 indicates the distribution of the responses to the Organisational Constraints questionnaire items, presented as percentages. Just over half of respondents indicated that conflicting job demands pose challenges on a daily basis, while interruptions, lack of information, inadequate help and equipment and supply issues were also problematic for many on a weekly or daily basis. The average score of 25.47 on the Organisational Constraints Scale is higher than the norm of 21.3, indicating a higher level of organisational constraints than average. A mean score of 20.96 was reported on the Quantitative Workload Inventory, which is higher than the available norm of 16.5, indicating a higher than average workload.

### 4.3 Regression Analyses

In order to determine the influence of personal and work‐related variables on QoWL, stepwise regression was conducted. Correlation between predictor variables in the model was first checked and indicated that a number of the predictor variables were correlated; however, most correlations were within the weak to moderate range. The only strong correlation observed was between work hours and full‐time/part‐time work status, *r* = −0.825, *p* < 0.001, reflecting that those who worked part‐time worked fewer hours. Follow‐on checks of variance inflation factors (VIF) were conducted and indicated that multicollinearity among the predictor variables was not significant, with VIFs ranging from 1.024 to 1.079. See Supporting Information: Table A1 in the Appendix where all VIFs are presented. Predictor variables in the model were entered in three blocks, with the personal variables entered in the first block (age, marital status, children), followed by work‐related factors (job permanence, full‐time/part‐time status, years’ work experience, hours per week), and with scores on the Quantitative Work Inventory and Organisational Constraints scale entered in the third and final block. Only age, job permanence, organisational constraints and quantitative workload were included following the stepwise process, with all other variables excluded from the analysis. A final model which explained 46.7% of the variance (Adjusted *R* ^2^ = 0.467) was found to be significant, *F*(4,104) = 21.904, *p* < 0.001. See Supporting Information: Table A1 (Appendix) for the results of the regression analysis, including the improvement in model fit with each step. Partial regression plots (Figures A1 and A2) are also presented in the Appendix for the significant predictor variables.

A second stepwise regression model examining the influence of the same predictor variables on the WHO‐5 mental well‐being scores was conducted. Predictor variables in this model were also entered in three blocks, with the personal variables entered in the first block (age, marital status, children), followed by work‐related factors (job permanence, full‐time/part‐time status, years’ work experience, hours per week), and with scores on the Quantitative Work Inventory and Organisational Constraints scale entered in the third and final block. All of the demographic factors, work‐related factors and Quantitative Workload Inventory scores were excluded during the stepwise regression process. See Supporting Information: Table A2 (Appendix) for the results of the regression analysis. A significant model was achieved, *F*(1,103) = 20.269, *p* < 0.001, accounting for 16.4% of the variance (Adjusted *R* ^2^ = 0.164). Organisational Constraints was the only significant predictor variable, *B* = −0.170, *B* SE = 0.038, *β* = −0.406. Table 3 presents a summary of the multiple linear regression analyses for both of the outcome variables, WrQoL and well‐being, including the standardised regression coefficients for the variables included in the final model and the significance levels.

**TABLE 3:** Multiple linear regression analyses for the outcome variables WrQoL and well‐being.

Variable	WrQoL		Well‐being	
	*β* (standardized beta value)	*p*	*β* (standardized beta value)	*p*
Age	−0.141	0.059	—	—
Job permanence	0.067	0.378	—	—
Quantitative Workload	−0.167	**0.026**	—	—
Organisational Constraints	−0.591	**< 0.001**	−0.406	**< 0.001**

*Note*: Boldface type indicates statistically significant values.

In the final analysis, linear regression was conducted to determine whether QoWL influenced turnover intentions. Using the enter method, a significant model emerged: *F*(1,103) = 31.502, *p* < 0.0005. The model explained 22.7% of the variance (Adjusted *R* ^2^ = 0.227), *B* = −3.207, *B* SE = 0.571, *β* = −0.484. See Supporting Information: Table A3 (Appendix) for the results of the regression analysis.

## 5 Discussion

The aim of this study was to characterise the QoWL and well‐being of SLTs working in Ireland and to determine the impact of personal variables (age, marital status, children) and work‐related variables (job permanence, full‐time/part‐time status, years’ work experience, sector, hours per week, workload, and organisational constraints) on both QoWL and well‐being. Regression models highlighted the significant relationship between work‐related variables and both QoWL and well‐being, and QoWL in turn was found to be significantly related to turnover intention. The low levels of well‐being and QoWL reported by participants in this study are of concern.

In response to the overall QoWL question, the mean score for participants in the current study was around the 20th percentile when compared to normative data (Easton and Van Laar 2018). QoWL was also slightly lower than in comparable studies (e.g., Zubair et al. 2017). However, both the normative data and the Zubair et al. (2017) findings relate to pre‐COVID QoWL, and, therefore, the comparison must be interpreted with caution. At the same time, our results are directly comparable to those reported by Hogan et al. (2023) in a recent post‐COVID examination of QoWL in occupational therapists in Ireland.

The other QoWL component scores were also all towards the lower end of the distribution. Notably, the mean scores on the General Well‐being (GWB), Job Career Satisfaction (JCS), and Working Conditions (WCS) components were all at the 30th percentile level when compared to the normative data for healthcare workers (Easton and Van Laar 2018). The mean scores for the Home‐Work Interface (HWI) component and the General Well‐being components were only slightly higher, within the 30th to 40th percentile range. In comparison to the scores reported by Hogan et al. (2023) for Irish occupational therapists, SLTs in this study reported lower scores on all seven components of QoWL.

At the most extreme end of the scale, the mean Stress at Work (SAW) score was only in the 20th percentile when compared to published norms (Easton and Van Laar 2018). In addition, as displayed in Figure 1, several organisational constraints frequently impacted on large proportions of participants in the current study. For example, conflicting work demands, interruptions, and organisational rules/procedures made work difficult for participants on a daily basis. The experience of such constraints can interfere with work and can be considered akin to work‐related stressors, with the potential to increase strain, lower well‐being, increase counterproductive work behaviour, and physical symptoms (Pindek and Spector 2016).

In the context of regression modelling, high workload and high organisational constraints were both significantly related to lower QoWL scores. The average score for participants on the Organisational Constraints measure is higher than the available norm, indicating a higher level of organisational constraints than in other studies. The majority of SLTs in this study worked in the public sector, which is similar to the profile of the occupational therapists in the Hogan et al. (2023) study. The mean Organisational Constraints scores in both studies are also comparable. Interestingly, Hogan et al. (2023) also reported that high organisational constraints and high workload predicted low QoWL scores in occupational therapists. These findings suggest that both of these groups of Irish allied healthcare practitioners in the public sector (i.e., both SLTs and OTs) are impacted by work conditions that inhibit or interfere with work.

Organisational constraints not only predicted QoWL but were also the only significant predictor of well‐being in this study. Specifically, higher organisational constraints predicted lower well‐being. Pindek and Spector (2016) previously conducted a meta‐analysis examining organisational constraints and its relationship with other variables and concluded that organisational constraints constitute a unique stressor, which is predictive of employee strain even when other potential stressors are controlled for. Müller et al. (2023) reported on the negative impact of the COVID‐19 pandemic on SLT services and provision in Ireland and have called for sustained support for the SLT profession and services to alleviate the build‐up of pressure on services. In particular, the results from this study suggest that the organisational constraints faced by many staff on a day‐to‐day basis require immediate attention. Conflicting work demands in particular were frequently reported as problematic, with just over half of respondents reporting it as a daily occurrence.

Notwithstanding the significant influence of organisational constraints on QoWL and well‐being, quantitative workload was also a significant predictor in the QoWL regression model. The average score on the Quantitative Workload Inventory in this study was higher than the published norm, indicating a heavy workload amongst respondents. Ewen et al. (2021) note that workload or caseload is a commonly cited factor in studies examining stress, job satisfaction, and burnout in SLTs. However, most studies to date have been conducted in the United States; therefore, this study provides new data and broadens the scope of understanding. Müller et al. (2023) have reported that the COVID‐19 pandemic significantly impacted service provision by SLTs in Ireland, and that the already over‐stretched services have been experiencing a post‐pandemic demand versus capacity issue as a result.

The WHO‐5 well‐being score of 46.05 in this study was below the cut‐off score of 50, which denotes low levels of well‐being in the study sample. Ewen et al. (2021) have noted in their review of well‐being, job satisfaction, stress, and burnout in SLTs that only one previous study has examined well‐being in SLTs; therefore, the findings of this study add to the limited literature in the area. However, more recently, the RCSLT (2022) have examined well‐being in SLTs and reported that the well‐being of SLTs in the United Kingdom was negatively impacted as a consequence of the COVID‐19 pandemic and its impact on services. Unfortunately, the well‐being findings from the RCSLT study cannot be directly compared to previous studies due to the use of different well‐being measures. Nevertheless, because of the use of the widely employed WHO‐5 measure of well‐being in this study, our findings can be compared to national‐level scores and to comparable allied healthcare professionals, where WHO‐5 scores are available. The average score reported in this study compares unfavourably with the average score reported in the national Green COVID survey conducted in Ireland (Guzman et al. 2020) and with the average score recently reported in a QoWL study conducted with occupational therapists in Ireland (Hogan et al. 2023).

Turnover intention in the current study sample was high, with just under three quarters of respondents indicating that they had considered resigning from their jobs. However, just under one third of respondents indicated that they were actively seeking other positions. While Ewen et al. (2021) identified a variety of factors that impact job retention in SLTs, including salary, workload, lack of job satisfaction and low job security, our findings indicate that lower QoWL is also a factor related to higher turnover intention among SLTs. Yeoh et al. (2024) acknowledge that attrition from allied healthcare professions remains challenging and note that innovative thinking, involving co‐design of solutions with, for and by allied healthcare professions, will be vital to address this issue. Qualitative studies examining QoWL and turnover among SLTs may provide further useful insight and build upon these initial quantitative findings. There are high vacancy rates for SLTs in Ireland, as high as 43% in Children's Disability Services (Health Service Executive 2023), and a recent survey indicated that some families report a wait of two years or more for SLT services (Sensational Kids, 2025). These reports highlight the urgent need to retain and grow the number of SLTs working in Ireland. As noted in the introduction, there have been challenges in developing a unified approach to delivering disability health services (HSE 2023), with IASLT members reporting systemic failures that are placing children and SLTs at risk, which in turn impact staff retention and recruitment in disability services.

Our study findings have implications for both policy and practice and may help to inform the ongoing development of unified approaches to health service delivery in Ireland. In the face of labour shortages and increased workload due to the COVID‐19 pandemic (Müller et al. 2023), healthcare employers not only have to focus on the recruitment of additional staff but also examine the working conditions and constraints faced by staff that may affect retention. We support the recommendations made by both the RCSLT and Müller et al. (2023) following their reviews of the impact of the COVID‐19 pandemic on speech and language therapy services in the United Kingdom and Ireland, respectively. In particular, we agree that sustained investment and support will be required going forward to ensure that adequate SLT services are provided, with effective strategies around recruitment and retention needed, as well as sustained support for the psychological well‐being of the SLT workforce (Müller et al. 2023; RCSLT 2022). In Ireland, an objective of the IASLT's 2023–2025 Strategic Plan focuses on leading, supporting, and protecting members of the profession (IASLT 2023). The IASLT has also committed to lobbying relevant government offices and agencies on key issues perceived by members to be career limiting (IASLT 2023). Quality of working life falls within this remit. Therefore, the IASLT has a key role to play in protecting and advocating for improved quality of working life for its members in the short and long term.

There is a dearth of literature examining QoWL in the allied healthcare professions, with a particular lack of research focused on SLTs. The findings of the current study provide some much‐needed insight into the work conditions experienced by SLTs in Ireland and their levels of well‐being. This study is timely and has addressed Ewen et al.'s (2021) call for research to examine the impact of individual and work‐related factors on the well‐being of SLTs. A larger scale nationally representative study or comparative international study may be of benefit to continue the focus on work conditions and challenges faced by SLTs, given the pressure on health services across the world currently.

## 6 Limitations

A number of limitations must be acknowledged. Although the response rate to the survey was higher than some of the other published studies with allied healthcare professionals (for example, Gupta et al. (2012) achieved a response rate of 2.9%), the response rate (10%) was low, and thus the findings may not be fully reflective of the SLT community in Ireland. At the same time, the response rate was similar to studies with other allied healthcare professionals; for example, Hogan et al. (2023) achieved a response rate of 6% in their study with occupational therapists. The current study sample was also fully female, and thus the findings may not be representative of the views of male SLTs. However, according to CORU (2021), the profession in Ireland is largely female‐dominated, with 98% female practitioners registered with CORU. Also, given that the majority of respondents to the survey were employed in the public sector, the findings may not reflect QoWL or well‐being within the private sector. Finally, the measure of turnover intention employed in this study does not differentiate between leaving one's current job, intention to leave the profession altogether, or moving from public to private practice or vice versa. Future studies should employ more nuanced measures of turnover intention (see Engström et al. 2023, for example) in order to more fully examine the implications of turnover for the SLT workforce.

## 7 Conclusion

The findings from this study paint a worrying picture of the well‐being and QoWL of SLTs in Ireland, with two key work‐related factors, organisational constraints and workload, identified as particularly problematic. Further research in the form of a larger‐scale examination of exposure to occupational stressors and barriers impeding quality of working life is required.

## Ethics Statement

Ethical approval for this study was granted by the University of Galway, Research Ethics Committee (Reference Number: 2022.03.007, March 2022).

## Consent

Patients were not involved in this study, therefore, non‐applicable.

## Conflicts of Interest

The authors declare no conflicts of interest.

## Supporting information




**Supporting Table A1**: Stepwise regression results for Quality of Working Life. **Supporting Table A2**: Stepwise regression results for well‐being. **Supporting Table A3**: Regression results for turnover intention. **Supporting Figure A1**: Partial regression plot demonstrating the relationship between work‐related quality of life and organisational constraints. **Supporting Figure A2**: Partial regression plot demonstrating the relationship between work‐related quality of life and quantitative workload.

## Data Availability

Data is available upon request.
