# Supplementary material for: Work‐Related Quality of Life and Well‐Being of Speech and Language Therapists in Ireland
Source: Int J Lang Commun Disord. 2025 Jul 25;60(5):e70090. doi: 10.1111/1460-6984.70090 (PMC12291614; doi:10.1111/1460-6984.70090)
Supplement: Supplementary file 1 — Supporting Table A1: Stepwise regression results for Quality of Working Life. Supporting Table A2: Stepwise regression results for well‐being. Supporting Table A3: Regression results for turnover intention. Supporting Figure A1: Partial regression plot demonstrating the relationship between work‐related quality of life and organisational constraints. Supporting Figure A2: Partial regression plot demonstrating the relationship between work‐related quality of life and quantitative workload. [file JLCD-60-0-s001.docx]

Appendix

**Table A1:** Stepwise regression results for Quality of Working Life

|  | **Variable** | **B** | **SE B** | **β** | **Significance** | **VIF** |
| --- | --- | --- | --- | --- | --- | --- |
| **Model 1** | Age | -.150 | .067 | -.214 | .028 | 1.000 |
| **Model 2** | Age | -.127 | .067 | -.182 | .059 | 1.024 |
|  | Job permanence | .392 | .180 | .208 | .032 | 1.024 |
| **Model 3** | Age | -.102 | .053 | -.146 | .056 | 1.028 |
|  | Job permanence | .160 | .145 | .085 | .271 | 1.067 |
|  | Organisational Constraints | -.048 | .006 | -.608 | <.001 | 1.051 |
| **Model 4** | Age | -.099 | .052 | -.141 | .059 | 1.029 |
|  | Job permanence | .126 | .143 | .067 | .378 | 1.079 |
|  | Organisational Constraints | -.047 | .006 | -.591 | **<.001** | 1.061 |
|  | Quantitative workload | -.017 | .007 | -.167 | **.026** | 1.029 |
|  |  |  |  |  |  |  |
|  | **R square** | **Adjusted R square** | **No of observations** |  |  |  |
| **Model 1** | .046 | .037 | 104 |  |  |  |
| **Model 2** | .088 | .070 | 104 |  |  |  |
| **Model 3** | .440 | .423 | 104 |  |  |  |
| **Model 4** | .467 | .446 | 104 |  |  |  |

Note: B = unstandardized beta value. SE = Standard Error, β = standardized beta value; VIF = variance inflation factor. Boldface type indicates statistically significant values in the final stepwise model (Model 4).

Figure A1: Partial regression plot demonstrating the relationship between work-related quality of life and organisational constraints.


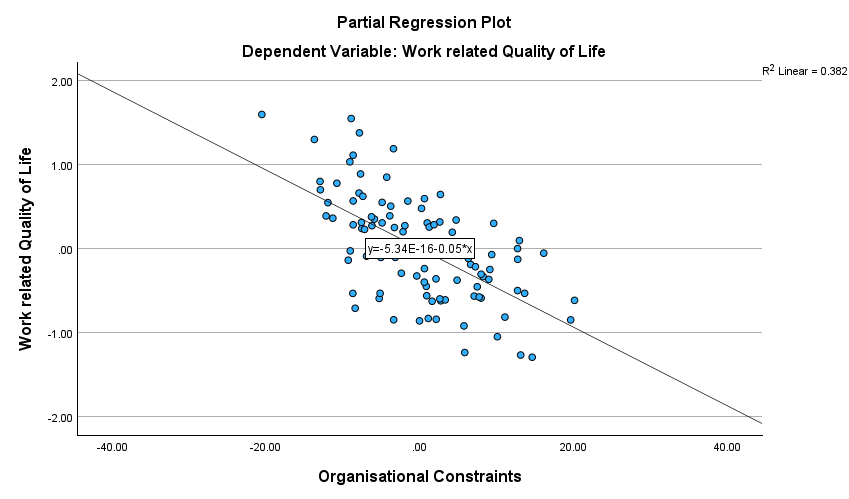


Figure A2: Partial regression plot demonstrating the relationship between work-related quality of life and quantitative workload.


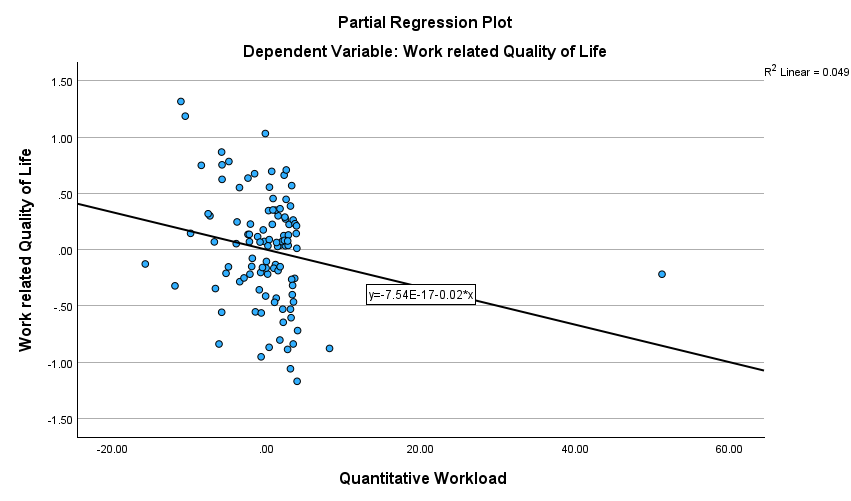


**Table A2:** Stepwise regression results for well-being

| **Variable** | **B** | **SE B** | **β** | **Significance** | **VIF** |
| --- | --- | --- | --- | --- | --- |
| Organisational Constraints | -.170 | .038 | -.406 | **<.001** | 1.000 |

Note: B = unstandardized beta value. SE = Standard Error, β = standardized beta value; VIF = variance inflation factor. Boldface type indicates statistically significant values.

**Table A3:** Regression results for turnover intention

| **Variable** | **B** | **SE B** | **β** | **Significance** | **VIF** |
| --- | --- | --- | --- | --- | --- |
| WrQoL | 3.207 | .571 | -.484 | **.000** | 1.000 |

Note: B = unstandardized beta value. SE = Standard Error, β = standardized beta value; VIF = variance inflation factor. Boldface type indicates statistically significant values.
